# c‐Abl Plays an Important Role in Mouse Preimplantation Embryo Development and the Dysregulation Associated With Decreased mTERT Expression

**DOI:** 10.1002/mrd.70039

**Published:** 2025-06-18

**Authors:** Ecem Yildirim, Tugce Onel, Aylin Yaba

**Affiliations:** ^1^ Department of Histology and Embryology University of Health Sciences, Faculty of Medicine İstanbul Turkiye; ^2^ Department of Histology and Embryology, Faculty of Medicine Demiroglu Bilim University Istanbul Turkiye; ^3^ Department of Histology and Embryology Yeditepe University Faculty of Medicine İstanbul Türkiye

**Keywords:** c‐Abl, mouse, mTERT, preimplantation embryo

## Abstract

c‐Abl encodes a cytoplasmic and nuclear protein tyrosine kinase that has been implicated in processes of cell growth, proliferation, differentiation, division, and regulation of cytoskeletal structure. mTERT is a catalytic subunit of mouse telomerase and it is very important for controlling cell proliferation and homeostasis by maintaining telomere length. We demonstrated before the interaction between c‐Abl and mTERT in mouse ovary and we suggested a role for c‐Abl in the regulation of telomerase function and proliferation in mouse granulosa cells. The current study aims to examine the c‐Abl and mTERT expression and potential interactions through mouse preimplantation embryonic development. To assess c‐Abl's function in embryonic development, siRNA‐mediated silencing of the *c‐Abl* was used in mouse preimplantation embryos. After siRNA transfection, the immunofluorescence was used to examine the c‐Abl pattern at embryonic development. Next, the levels of *mTERT* and *c‐Abl* mRNA were compared. The results show that c‐Abl is expressed in mouse preimplantation embryos at all developmental stages, with the cytoplasmic expression all through from the 2‐cell to the blastocyst. Additionally, c‐Abl is presented very intense expression in blastomer‐blastomer junctions. The siRNA‐mediated depletion of *c‐Abl* showed developmental abnormalities at the 8‐ to 16‐cell and morula to blastocyst and also with significantly decreased blastocyst development rate. Moreover, expression of the mTERT telomerase catalytic subunit was significantly reduced in *c‐Abl*‐depleted embryos during preimplantation embryonic development. Finally, we demonstrate that c‐Abl may play a crucial role in compaction and preimplantation embryo development, and that the relationship between c‐Abl and mTERT has developmental importance in early embryogenesis.

## Graphical abstract

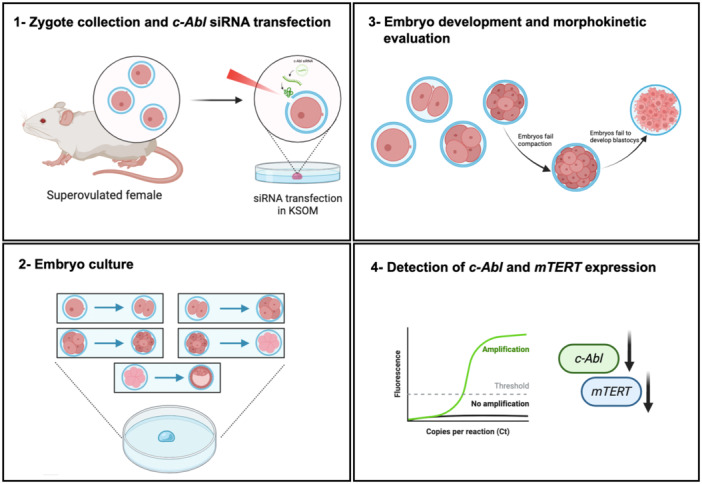

**Abbreviations**
Abl: abelson tyrosine kinase
BSA: Bovine serum albumin
hCG: human chorionic gonadotropin
HTF: human tubal fluid
IP: intraperitoneal
mTERT: mouse telomerase reverse transcriptase
PMSG: pregnant mare serum gonadotropin

## 1 Introduction

Preimplantation embryo development is the process that begins with the formation of the zygote following fertilization and is completed with the implantation of embryo (Bell and Watson 2013). The zygote formed after fertilization undergoes successive mitotic divisions and transitions to 2‐, 4‐, and 8‐cells, respectively. During the transition of mouse embryos from the 8‐cell to the 16‐cell (morula), a critical event is called compaction, and after compaction, the embryo reaches morula (Yildirim et al. 2021). In the morula, the blastomeres are connected via junctions and they present cellular polarity (Bruce and Zernicka‐Goetz 2010; Canse et al. 2023). This stage is very important for cell fate determination and cell specification. Compaction is an important morphogenetic process based on the mechanical interactions of cells and includes morphologic events with the surface tension of blastomeres, interblastomere angle, and adhesion forces (Turlier and Maître 2015; Canse et al. 2023). The period of early stages of embryogenesis are crucial to comprehend the molecular mechanisms involved in critical processes such as fertilization, cleavage, compaction, and lineage specialization.

Abelson tyrosine kinase (c‐Abl) is a member of the non‐receptor protein tyrosine kinase family (Laneuville 1995). c‐Abl is a cytoplasmic and nuclear protein tyrosine kinase that has been implicated in processes of cell growth, proliferation, differentiation, division, adhesion, regulation of cytoskeletal structure, and stress response (O'Neill et al. 1997; Plattner et al. 1999; Hantschel and Superti‐Furga 2004; Shaul and Ben‐Yehoyada 2005; Yaba et al. 2020; Yildirim and Yaba 2020a). c‐Abl is activated by DNA double‐strand breaks and the proteins involved in the repair of these lesions in telomere control (Kharbanda et al. 2000). Previous studies demonstrate that homozygous mutations in the *c‐Abl* gene are characterized by increased perinatal mortality and reduced fertility (Li et al. 2000), and defects in embryonic development (Hantschel and Superti‐Furga 2004; Hernández et al. 2004). We previously showed that c‐Abl regulates gene transcription during mouse estrus cycle (Ucar et al. 2012), embryonic and fetal development (Ahmad and Naz 1994; Yaba et al. 2011), and placental development (Yaba et al. 2011). Although some studies have reported that c‐Abl is expressed during early embryonic development, no study has investigated its expression characteristics and stage‐specific functions during early embryonic development. According to Ahmad and Naz, since particular antibodies against the c‐abl proto‐oncogene products specifically hindered embryonic development, notably from the morula to blastocyst, they may be implicated in the development of preimplantation embryos (Ahmad and Naz 1994). In a study conducted in 2017, it was shown that c‐Abl is expressed during preimplantation embryonic development (Mutluay and Oner 2017). Both studies investigated the expression of c‐Abl on mouse embryonic development. However, the stage‐specific functions of c‐Abl in preimplantation embryo development remain unclear.

Telomerase is a ribonucleoprotein complex that elongates telomeres, involved in several essential biological functions, and activation of telomerase is associated with successful embryonic development (Bekaert et al. 2004). mTERT (mouse telomerase reverse transcriptase) is a necessary subunit of telomerase complex in mice and its expression correlates with telomerase activity during the differentiation of embryonic stem cells (Armstrong et al. 2000), and maintenance of telomerase activity provides a means of preventing cellular senescence (Blackburn 1991; Montgomery et al. 2011). Telomerase activity is closely regulated during the early stages of embryonic development. Immature oocyte presents high telomerase activity. After fertilization, zygotes had lower and blastocysts had higher telomerase activity (Xu and Yang 2000; Wright 2001). It has been shown that telomere length is increased in human preimplantation embryos, and there are significantly shorter telomeres in the cleavage stages (Treff et al. 2011). The critical shortening of telomeres in human embryos causes failure of embryo implantation (Biron‐Shental et al. 2010; Biron‐Shental et al. 2011). Telomerase homozygous knockout mice showed telomere shortening, misalignment of metaphase chromosomes, and female infertility (Liu et al. 2004). We previously demonstrated the expression of c‐Abl and mTERT during mouse prenatal and postnatal gonadal development. We also showed the interaction of the mTERT telomerase catalytic subunit with the c‐Abl tyrosine kinase in mouse granulosa cells (Yaba et al. 2020). Considering these results, it can be said that telomerase both preserves genetic integrity and increases the survival chances of cells. The proper elongation of telomeres allows cells to proliferate with the correct mechanisms in place (Mori et al. 2024). In this study, we aimed to examine the stage‐specific expression and function of c‐Abl in early preimplantation embryo development and the potential relationship between c‐Abl and mTERT during the preimplantation embryonic period.

## 2 Materials and Methods

### 2.1 Experimental Animal Ethics Statements

All animals used in this study were obtained from the Yeditepe University Faculty of Medicine Experimental Research Center (YUDETAM) and all the experimental procedures have been approved by the Yeditepe University Ethical Committee. This manuscript complies with the ARRIVE guidelines (Percie du Sert et al. 2020). All procedures were carried out under the supervision of veterinarians. All experiments were performed in accordance with the guidelines and regulations of the Faculty of Medicine of Yeditepe University.

#### 2.1.1 Superovulation, Mouse Embryo Collection, and Embryo Culture

**Figure 1:**
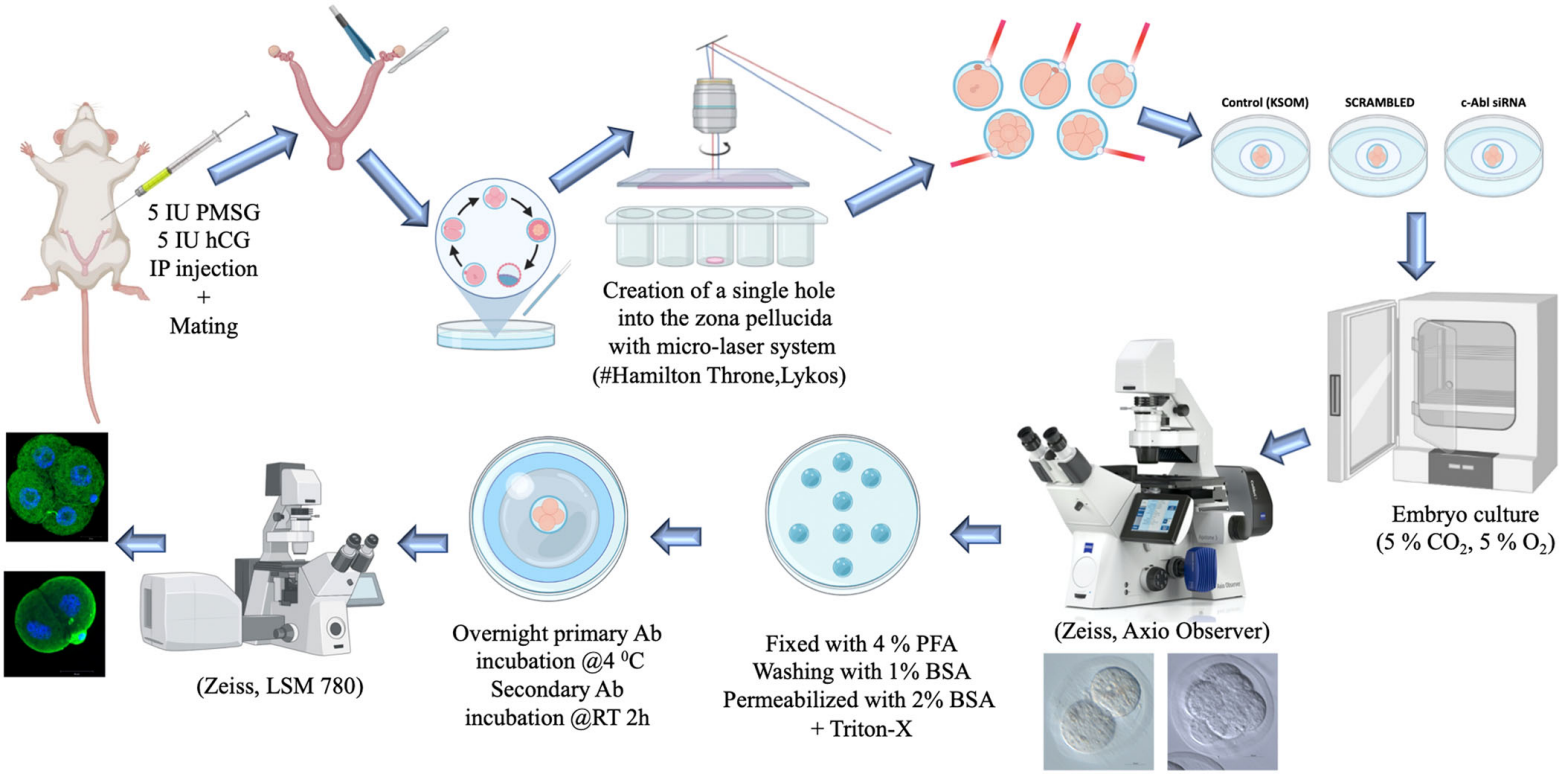
Graphical representation of the methods. (Artwork was initially created in BioRender).

Six‐week‐old female BalbC mice were superovulated by intraperitoneal (IP) injection of 7.5 IU pregnant mare serum gonadotropin (PMSG; Sigma‐Aldrich) followed 48 h later by 7.5 IU human chorionic gonadotropin (hCG; Sigma‐Aldrich) before mating with BalbC males. The next day, animals with vaginal plaque or sperm on the vaginal smear were considered mated. Embryos were collected from the oviduct at specified times following hCG injection, which corresponded to appropriate cleavage stages: zygote (1‐cell), 2‐cell, 4‐cell, 8‐cell, morula, and blastocyst were collected by uterus flushing. Embryos were collected into HTF (human tubal fluid) medium (Irvine Scientific) washed several times in flushing medium and collected in pools to be either: (1) in vitro culture for siRNA transfection; (2) fixed for immunofluorescence analysis of protein localization; (3) frozen and stored at −80°C for protein/RNA extraction. Isolated embryos were cultured in KSOM + AA incubation medium at 37°C containing 5% CO_2_ and 5% O_2_. Morphological evaluations of embryos were followed under a Zeiss Axio microscope before and after transfection (Figure 1).

#### 2.1.2 siRNA Transfection and In Vitro Culture

siRNA‐transfected embryos were cultured in KSOM medium at 37°C in a humidified atmosphere of 5% CO_2_ and 5% O_2_. The zona pellucida, a glycoprotein layer surrounding the mouse embryos, to facilitate the siRNA transfection from zona pellucida, just before the transfection procedure, a hole was made in the zona pellucida of the embryos with a Hamilton micro‐laser system. Afterwards, mouse embryos were incubated with 10 μM c‐Abl siRNA (ON‐TARGETplus Mouse Abl1 siRNA, mouse, #AI325092, HORIZON). The ON‐TARGETplus Mouse Abl1 siRNA we used consists of four different siRNA sequences. These sequences target different regions of the c‐Abl mRNA (Target Sequences: AGUAAGCGCUUCCUGCGUU, CGAAGCAGCUCGAUGGAAC, UGAGCUAUGUGGACUCUAU, GGAACCACCAUUCUACAUA), DharmaFECT transfection reagent (Thermo Scientific) and siGLO green transfection indicator (Thermo Scientific). 10 μM scrambled siRNA was used for c‐Abl siRNA control. These siRNA sequences are designed to cover the regions encoding the SH3 (Src Homology 3), SH2 (Src Homology 2), kinase, and C‐terminal domains of the c‐Abl gene (Hantschel and Superti‐Furga 2004). Thus, it ensures the suppression of all functional domains of the c‐Abl protein at the mRNA level. The effect of siRNAs is confirmed by the general inhibition of protein synthesis, rendering all functions of c‐Abl (e.g., protein‐protein interactions through the SH3 domain, binding to phosphorylated proteins via the SH2 domain, and substrate phosphorylation by the kinase domain) inactive. Embryos were cultured until they passed to the next stage (Figure 1).

#### 2.1.3 Immunofluorescence Analysis

Mouse preimplantation embryos were collected and processed for application of whole‐mount immunofluorescence as described (Sozen et al. 2015). Embryos were washed in 1% BSA in PBS (0.1 M, pH 7.4) and then fixed in 4% paraformaldehyde in PBS, washed 1% BSA in PBS. The fixed embryos were permeabilized and blocked by incubating in 2% BSA in PBS with %0.01 Triton X‐100 at room temperature for 1 h. The embryos were then washed three times in 1% BSA in PBS followed by the incubation of embryos with rabbit polyclonal c‐Abl antibody (#ab15130, Abcam, 1:100) and mTERT antibody (#PA5‐11446, Thermo Scientific, 1:100) in blocking solution at 4°C overnight. Embryos were then washed three times with 1% BSA and incubated with 594 goat anti‐rabbit secondary antibodies (#ab150080, Abcam, 1:750 for 1.5 h) in a blocking solution at room temperature. Finally, for nuclei staining, the DAPI mounting medium was diluted with PBS to form a drop, and embryos were placed in this drop; imaging was performed under a confocal microscope (Zeiss, LSM780) (Figure 1).

### 2.2 RNA Isolation and qRT‐PCR

Total RNAs were isolated from the preimplantation embryos by using an RNA Purification Kit (#PP210S, Jena Bioscience). Complementary DNA (cDNA) reaction was carried out using the SCRIPT cDNA Synthesis Kit (#PCR511S, Jena Bioscience) according to the manufacturer's instructions. Specific primer pairs used for RT‐PCR (c‐Abl‐ F 5′‐CGG GAC CAT GTT GGA GAT‐3′, R 5′‐TTC ATA CCG CAG CGA GATG‐3′, mTERT‐ F 5′‐CCTGCGGCCCATTGTGAAC‐3′, R 5′‐GTG GACTTG GCC TTG GCT ATC TCT‐3′ and Beta‐actin‐ F 50‐GACCTCTATG‐ CAACACAGT‐30, R 50‐TTG CTG ATC CAC ATC TGCT‐30). qRT‐PCR reaction was set up in a volume of 12 μL containing 9 μL of Maxima SYBR Green qPCR Master Mix (2x), 1 μL forward, 1 μL reverse for primers, and 1 μL cDNA was performed. The specificity of qRT‐PCR was confirmed by melting curve analysis for each reaction. (The reaction started at 95°C for 3 min. Then, the reaction was continued for 55 cycles at 95°C 15 s., 47°C 30 s., 55^o^C 30 s.). All expression data were normalized by dividing the amount of the target gene by the amount of Beta‐actin used as an internal control.

#### 2.2.1 Statistical Analysis

Morphological development data were analyzed with 2‐way ANOVA (for ZP thickness) and One‐way ANOVA (used Geisser‐Greenhouse correction). The immunofluorescence intensity results and qRT‐PCR results were analyzed by using ImageJ, and statistical analysis was performed with GraphPad Prism. Normally distributed data were analyzed by a Two‐Way ANOVA test to compare multiple groups. Differences were considered significant when *p* < 0.05.

## 3 Results

### 3.1 siRNA‐Mediated c‐Abl Depletion Affects Embryo Development and Alters Embryo Morphokinetics During the Preimplantation of Mouse Embryos

**Figure 2:**
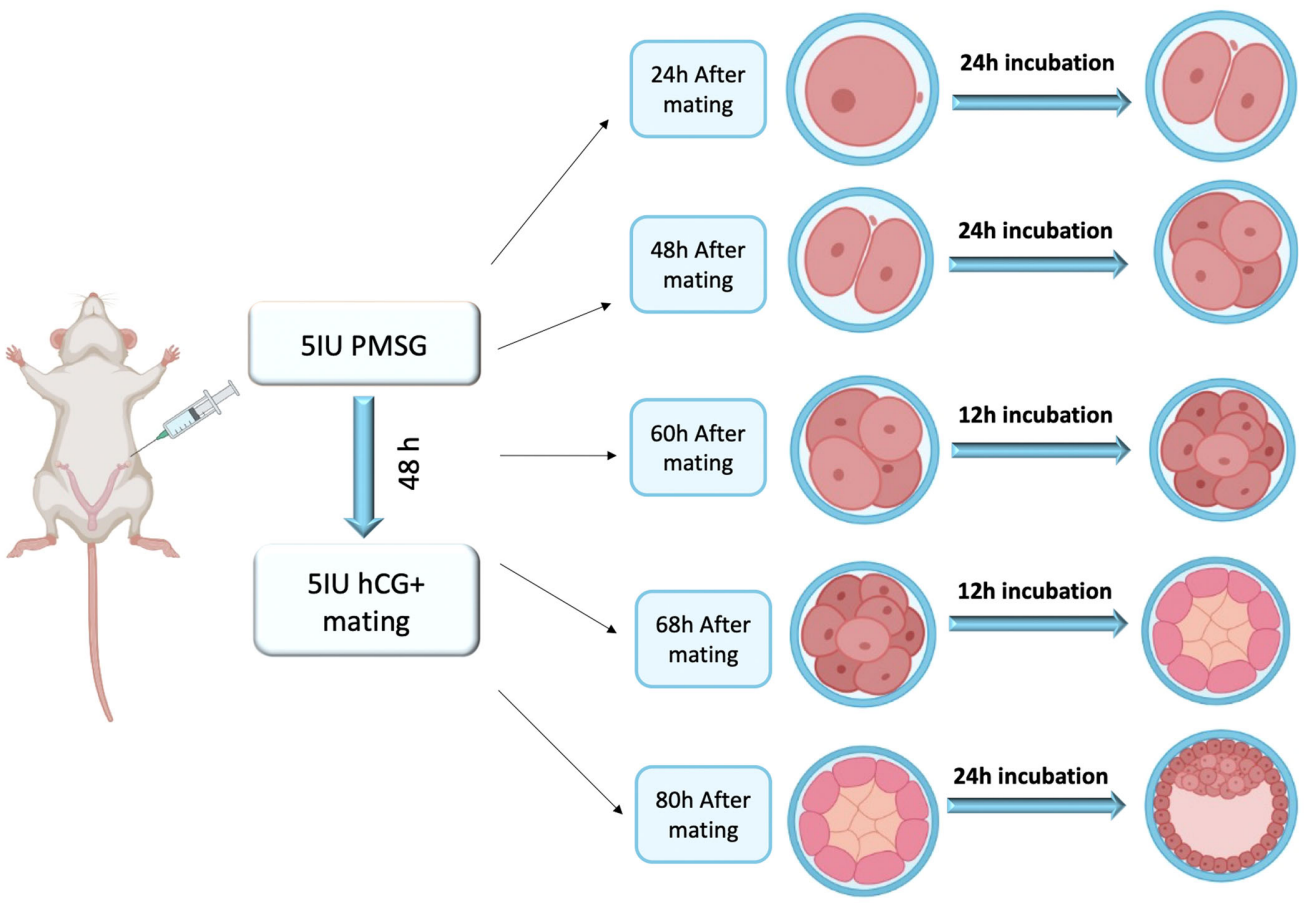
A graphical representation of the stages and times at which embryos collected from the oviduct were isolated for embryo culture. Zygote, 2‐, 4‐, 8‐cell, and morula embryos were collected and cultured with c‐Abl siRNA until they passed to the next stage (Artwork was initially created in BioRender).

The zygote, 2‐, 4‐, 8‐cell, and morula stage embryos were transfected, respectively, with siRNA against c‐Abl and cultured to 2‐, 4‐, 8‐cell, morula, and blastocyst, respectively, to show the involvement of c‐Abl in early mouse embryonic development. Abl siRNA was used to culture zygote, 2‐, 4‐, 8‐cell, and morula embryos up until they reached the subsequent stage (Figure 2). There were three groups: a control group with no transfection, a c‐Abl siRNA‐transfected group, and a scrambled siRNA‐transfected group. Developmental potentials of embryos transfected with c‐Abl siRNA were compared with control and scrambled embryos. Control, scrambled and c‐Abl‐depleted embryos were morphologically identical during the transition from zygote to 2‐cell, 2‐cell to 4‐cell, and 4‐cell to 8‐cell. In control group embryos, the borders of the blastomeres disappeared, and blastomeres were morphologically indistinguishable in morula. However, in c‐Abl‐depleted morula stage embryos, blastomeres were distinguishable at the transition to the 8‐cell‐morula.

**Figure 3:**
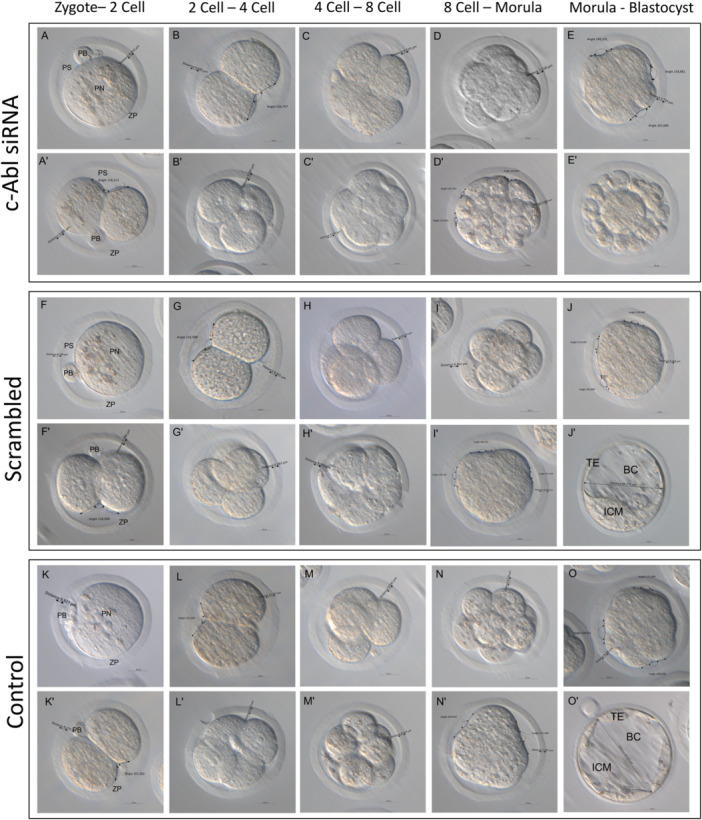
Morphological evaluation of cultured mouse embryos. The developmental potentials between control, Scrambled, and c‐Abl siRNA applied groups were compared morphologically. Each group was evaluated in order according to their developmental rate (from zygote to 2‐cell, from 2‐cell to 4‐cell, from 4‐cell to 8‐cell, from 8‐cell to 16‐cell‐morula, from Morula to blastocyst).

**Figure 4:**
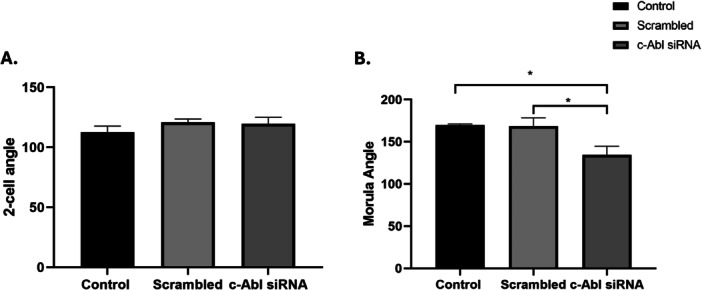
The evaluation of morphological development of embryos, (A) 2‐cell angle and (B) morula angle (*p < 0.1).

**Figure 5:**
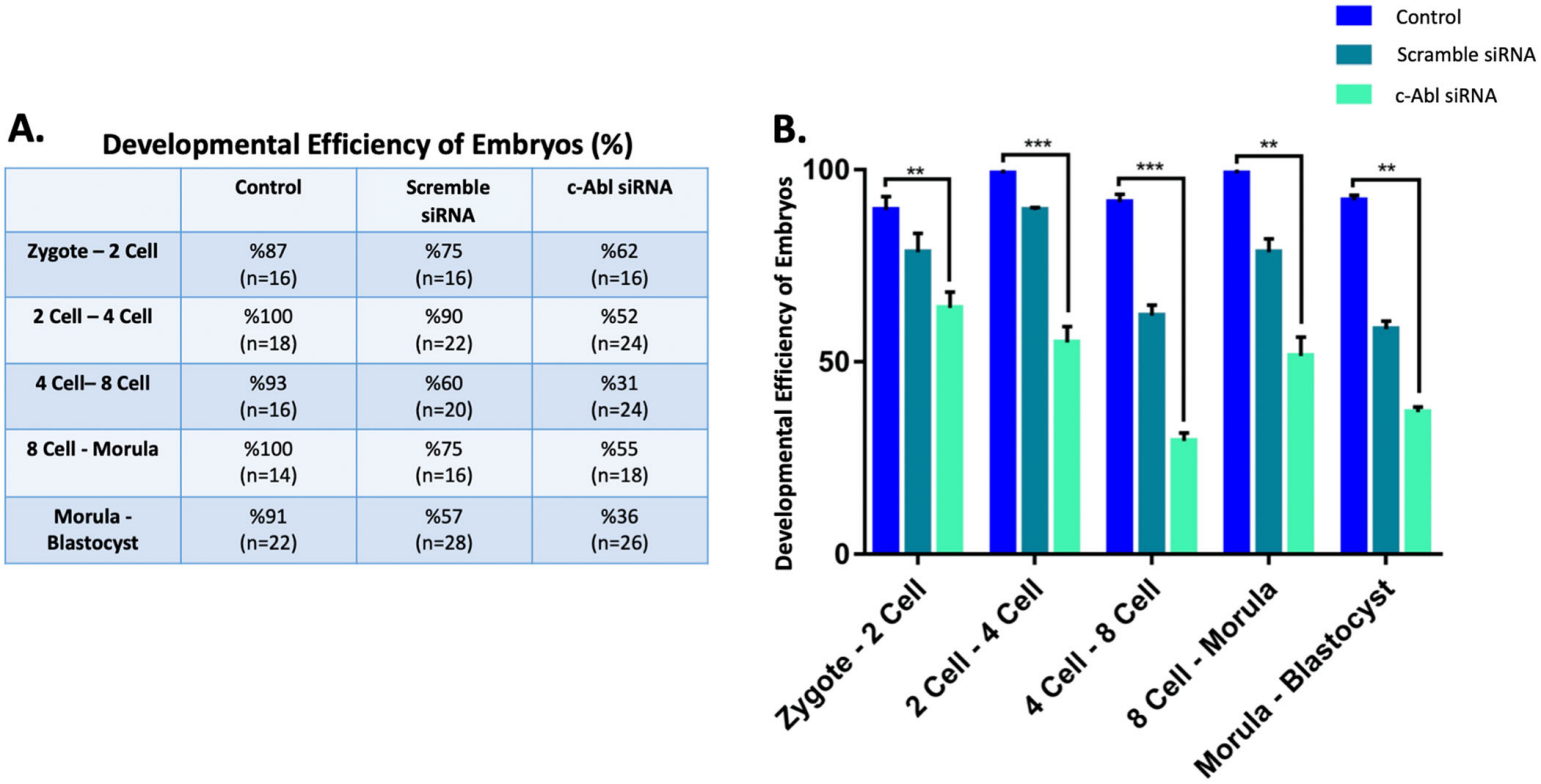
(A) Statistical evaluation of development rates of embryos. Determination of the development rate of embryos in control, scrambled and c‐Abl siRNA groups as percentage. The number of embryos used for each group is shown. (B) When the embryos in the c‐Abl siRNA group were compared with the control group, it was observed that the embryos developing in the c‐Abl siRNA group decreased significantly, especially in the 4‐cell‐8‐cell transitional stage. However, the development rate was significantly decreased in the c‐Abl siRNA group in the 8‐cell‐morula transition and morula‐blastocyst transition (**p < 0.01, ***p < 0.001).

The angle between two neighboring blastomeres, a crucial sign of the embryo's compaction, was measured and quantified (Turlier and Maître 2015; Giammona and Campàs 2021). siRNA‐mediated knockdown of c‐Abl with reduces a possible natural rearrangement of the blastomeres and prevents the successful development of the embryo to the blastocyst (Figure 3). In the c‐Abl scrambled group, the morphology and developmental potential of embryos were similar to the control group (Figure 2). c‐Abl‐depleted embryos did not show a difference for 2‐cell angle in comparison to control groups. However, it was determined that the angle was significantly acute in the c‐Abl siRNA transfection group compared to the control and scrambled groups (Figure 4A). Compacting blastomeres in the control group embryos showed increasing inter‐blastomere angle, and the morula stage embryos became smoother as a result of blastomeres adhering more tightly to each other. The rounder cells in uncompacted embryos at the morula demonstrated reduced inter‐blastomere angles between neighboring blastomeres, compared to c‐Abl‐depleted embryos. The blastomeres did not start to compress tightly, and there was no apical surface elongation (Figure 4B). In addition to the morphological comparison, the development rate of the c‐Abl‐depleted embryos significantly reduced in comparison to control and scrambled group embryos (Figure 5).

### 3.2 Immunofluorescence was Used to Identify c‐Abl in Preimplantation Embryos and Examine Its Localization Pattern

**Figure 6:**
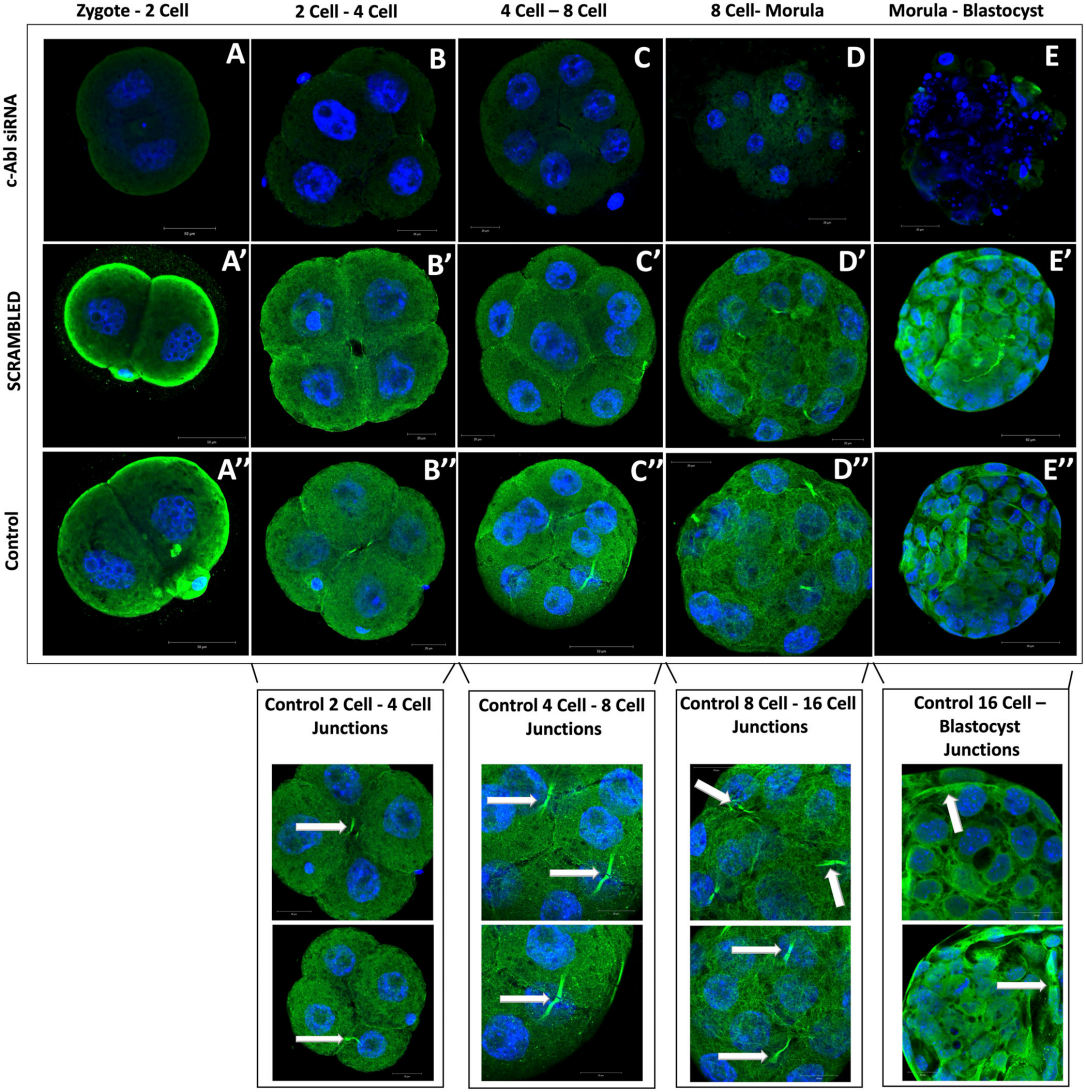
Determination of c‐Abl localization by immunofluorescence method in pre‐implantation embryo development. c‐Abl knockdown in preimplantation embryos using siRNA against c‐Abl mRNA. c‐Abl knockdown was confirmed by indirect immunofluorescence and the localization pattern of c‐Abl was analyzed in control, Scramble, and c‐Abl siRNA embryos. A cross‐section of representative embryos through the equatorial plane shows the localization of c‐Abl in the nuclei of the trophectoderm in all groups. A magnified image of the dashed area is shown on the right. Especially in the 4‐cell – 8‐cell, the connections associated with c‐Abl are seen in the control group. Successful compaction could not occur in the c‐Abl siRNA group. As a result, a healthy morula and blastocyst could not be formed in the c‐Abl siRNA group. Scale Bars = 20 μm and 50 μm (for the magnification). Representative images from *n* = 8 control, *n* = 10 scrambled siRNA‐transfected embryos, and *n* = 10 c‐Abl siRNA‐transfected embryos across three independent experiments.

Immunofluorescence was used after the embryo culture to detect the expression of c‐Abl in all experimental groups and to demonstrate that c‐Abl is depleted and not expressed in mouse embryos at all stages of preimplantation development (Figure 6).

**Figure 7:**
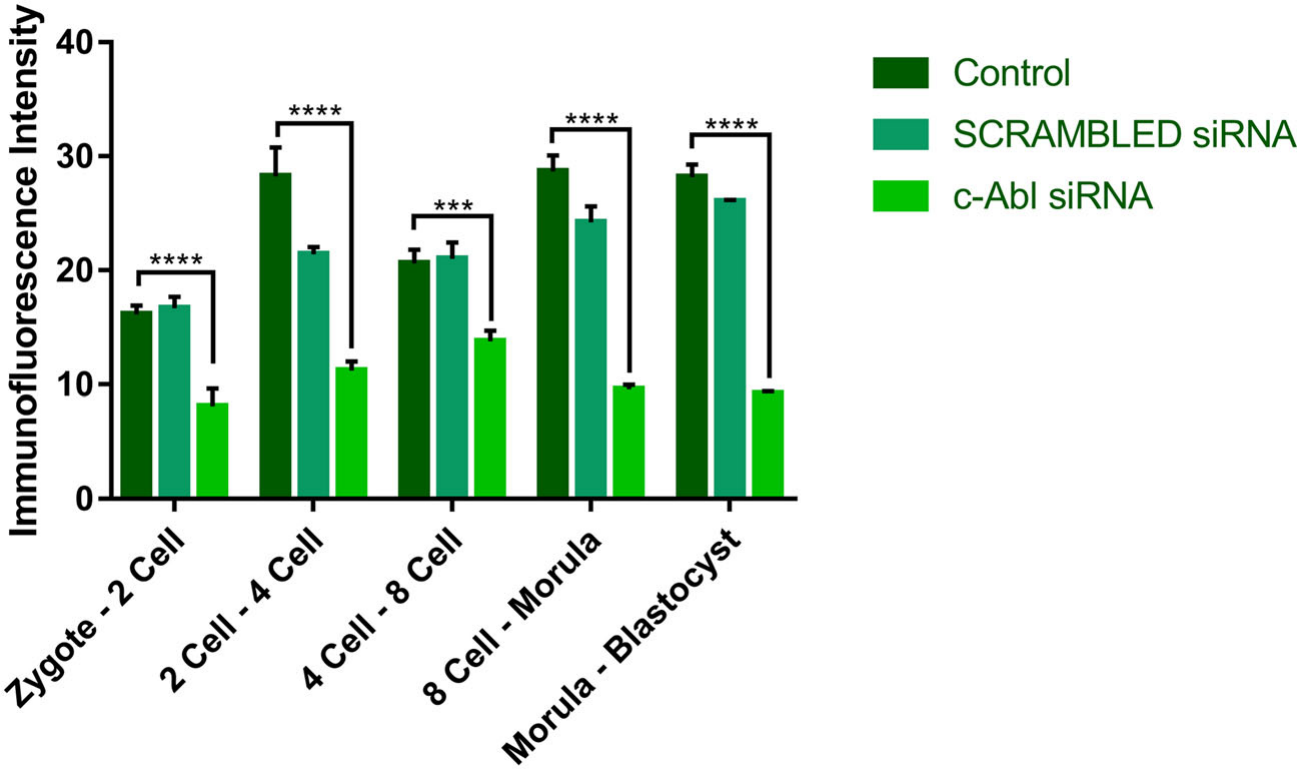
Comparison of c‐Abl Immunofluorescence intensity between groups. Comparing the immunofluorescence intensity, it was seen that the c‐Abl expression intensity in the control group was significantly higher than in the c‐Abl siRNA transfection group (*p < 0.5).

In the control and the scrambled group, c‐Abl displayed cytoplasmic and subcortical expression during the stage of transition from the zygote to 2‐cell. However, in the c‐Abl siRNA transfection group, c‐Abl expression was unable to be detected (Figure 6A‐A′‐A″). During the transition of the 2‐cell to 4‐cell stage, c‐Abl localized in the cytoplasm and intercellular region in control and scrambled embryos. However, in c‐Abl siRNA transfection embryos, c‐Abl showed very weak expression in the intercellular area and there was no cytoplasmic expression (6B‐B′‐B″). In the transition to 4‐cell to 8‐cell, c‐Abl showed cytoplasmic expression and intense expression in the intercellular area in control and scrambled groups. c‐Abl expression was not observed in the c‐Abl siRNA transfection embryos (6C‐C′‐C″). In the transition of 8‐cell to morula, which is the stage where compaction occurs, we observed that the control group and scrambled group embryos successfully compacted and both group embryos showed c‐Abl expression in the intercellular area. However, there is no c‐Abl expression in c‐Abl‐depleted embryos, and additionally c‐Abl siRNA transfection embryos showed failure to compaction and were eventually arrested (6D‐D′‐D″). c‐Abl expression was observed in the cytoplasm and intercellular region in the control and scrambled‐siRNA transfected embryos, particularly in TE cells and both groups of embryos showed development until blastocyst stage. We observed that c‐Abl‐depleted embryos did not reach the blastocyst stage because compaction did not occur (6E‐E′‐E″). The fluorescence intensities of the control, scrambled, and c‐Abl siRNA groups were examined, the c‐Abl‐depleted embryos showed lower expression than that of the control and scrambled groups (*p < 0.5) (Figure 7).

### 3.3 The Expression of *mTERT* is Downregulated by *c‐Abl* Depletion

**Figure 8:**
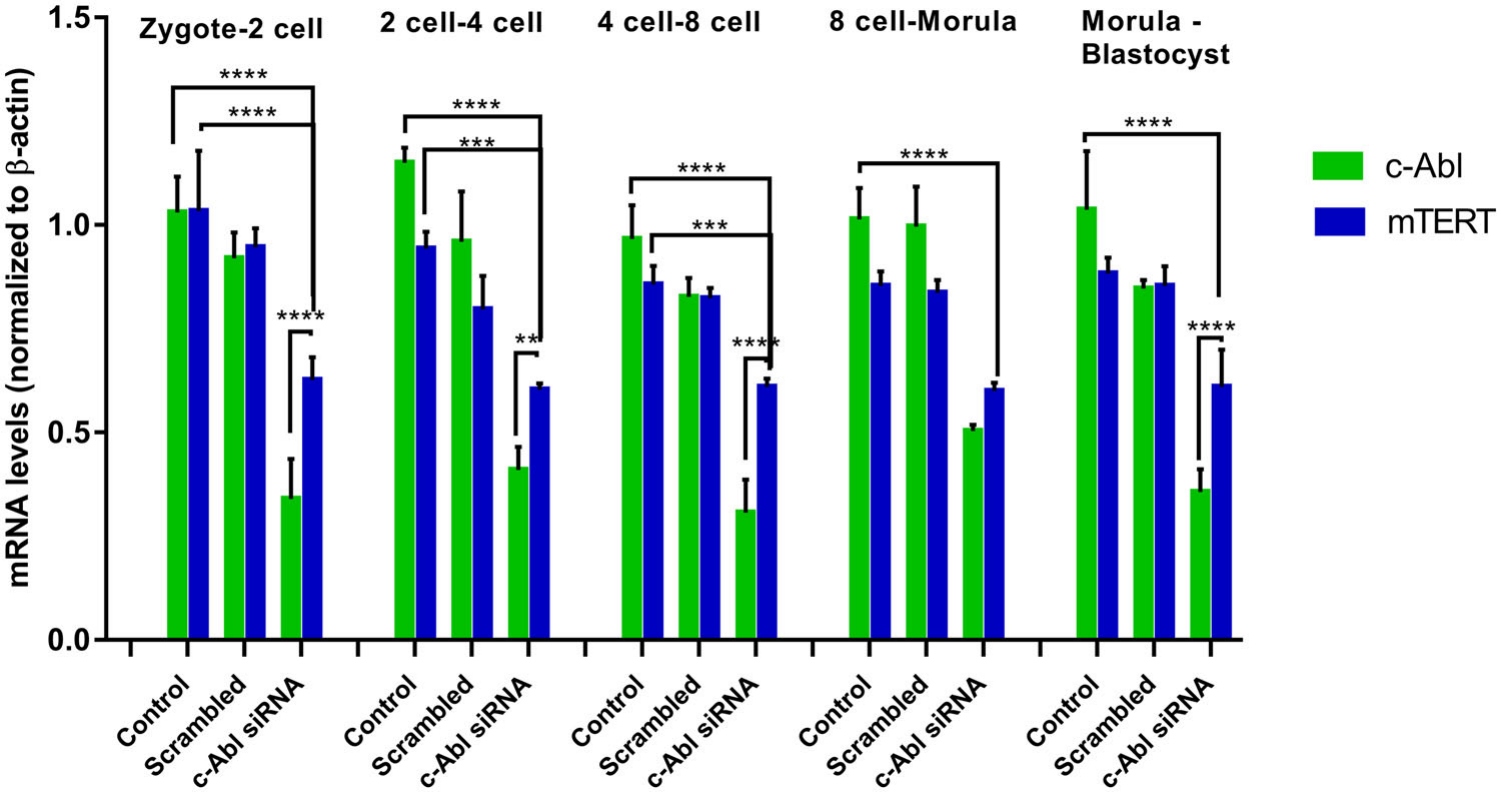
Comparison of *c‐Abl* and *mTERT* levels at the mRNA level in preimplantation embryos in which c‐Abl is silenced. c‐Abl mRNA level was significantly decreased in the c‐Abl siRNA transfection group in which c‐Abl was silenced compared to the control group. mTERT levels in the c‐Abl silenced groups were significantly reduced compared to the mRNA level in the control groups. It was observed that mTERT mRNA levels decreased significantly when c‐Abl was silenced. The *mTERT* mRNA level showed a similar decrease as the c‐Abl siRNA level (**p < 0.01, ***p < 0.001, ****p < 0.0001).

Quantitative real‐time PCR was used to analyze the effects of *c‐Abl* depletion on the expression of the telomerase activity‐related *mTERT* expression from the zygote to the 2‐cell, 4‐cell, 8‐cell, morula, and blastocyst stage during mouse preimplantation embryonic development. The embryonic development was evaluated at the transitional stage of zygote‐2 cell, 2‐cell‐4 cell, 4‐cell‐8 cell, 8‐cell‐morula, and morula‐blastocyst. *c‐Abl* expression is decreased in the groups in which c‐Abl is silenced, while *mTERT* expression is significantly decreased compared to the control group (Figure 8). The depletion of *c‐Abl* most affected the *mTERT* expression in the zygote to 2‐cell, 2‐cell to 4‐cell, and 4‐cell to 8‐cell embryo stage of early dvelopment. The identification of a correlation between the *c‐Abl* and *mTERT* gene mRNA levels could suggest that c‐Abl may be associated with a mechanism that can regulate mTERT expression.

## 4 Discussion

Here we report that impaired expression of *c‐Abl* tyrosine kinase‐related *mTERT* gene expression in mouse preimplantation embryos causes decreased blastomere‐blastomere angle with uncompacted phenotypes and developmental abnormalities. The degree of compaction is positively correlated with increased success of in vitro embryonic development. We showed that c‐Abl knockdown embryos failed to compaction and they were eventually arrested. We also showed the relationship between c‐Abl tyrosine kinase and mTERT is associated with a failure of cell fate specialization and determination during early embryonic development.

We sought to take into account the relationship between c‐Abl and mTERT in preimplantation embryonic development based on our study, which revealed an interaction between *mTERT* and *c‐Abl* in mouse ovarian granulosa cells and demonstrated that c‐Abl may play a role in the regulation of telomerase function and proliferation in mouse granulosa cells (Yaba et al. 2020). Telomere length in mouse preimplantation embryos has also been evaluated in research, and the results have demonstrated a considerable increase in telomere length from the 8‐cell through the morula and blastocyst (Turner et al. 2010). It has been reported that this increase in telomere length seen during the morula to blastocyst transition is due to telomerase activity (Schaetzlein et al. 2004). It became known that as preimplantation human embryos developed to the blastocyst, telomere lengthening (Turner et al. 2010). In addition, studies on human preimplantation embryos have shown that telomere length is reprogrammed by recombination in the early stages of embryonic development and is maintained by telomerase in the later stages of preimplantation development, even in some aneuploid embryos (Wang et al. 2023). Based on these studies, we carried out this study to ascertain the functional significance of c‐Abl during early embryogenesis and to shed light on the interaction between c‐Abl and mTERT during early embryo development. To show this, we used siRNA transfection to suppress c‐Abl at every stage of preimplantation embryo development to identify early embryonic developmental function and potential relation with mTERT.

Apico‐basal cell polarity has been suggested to be observed in blastomeres at the 8‐cell and subsequently plays a role in the differentiation of ICM and TE cells (Johnson 1981). In the inside‐outside model, which indicates that cell differentiation is based on its location; It has been suggested that the position of the blastomeres in the embryo, especially a noncontact surface region, forms the apical area and thus determines the cell fate (Korotkevich et al. 2017) (Yildirim and Yaba 2020a). TE cells have apical‐basal polarity and junctional complexes known as TJs and AJs that protect the blastocyst cavity (Eckert and Fleming 2008; Canse et al. 2023). It has been suggested that the ICM cell fate is determined at the 32‐cell (Tarkowski et al. 2010), the early‐intermediate blastocyst (~64 cells) (Suwińska et al. 2008; Stephenson et al. 2010), or the late blastocyst (Hogan and Tilly 1978; Grabarek et al. 2012). Among these stages, the polarization of blastomeres is regulated by cell‐cell connections, cytoskeletal structure, and the signaling pathways involved in these mechanisms (Yildirim et al. 2021). We aimed to address the failure of the preimplantation embryo to develop into a blastocyst when c‐Abl was silenced with siRNA, especially after the 8‐cell stage, and the potential importance of the molecular mechanism in compaction, polarity and further cell fate determination. In the 2‐cell, there was no apparent distinction in the morphology of the c‐Abl silenced and control groups. However, the loss of the c‐Abl pattern between blastomeres, particularly in the compaction (transition from 8‐cell to morula), supports the idea that the failure of compaction is the loss of c‐Abl localization between blastomeres. According to specific hypotheses, polarity may be disturbed when c‐Abl is silenced, preventing blastomeres in the morula from correctly establishing their character and compacting. The inability to establish polarization in the embryo, along with the lack of polarization in the blastomeres, may have prevented compaction, thereby hindering the progression to the blastocyst stage.

c‐Abl plays an important role in the regulation of the cell cycle. This protein particularly manages phosphorylation events during the G1/S transition, mitosis, and cell division. The absence of c‐Abl prevents cells from dividing normally and disrupts the proper functioning of the cell cycle (Pendergast 2002). Silencing of *c‐Abl* in preimplantation embryo development can lead to disruptions in cell division during the morula and blastocyst stages. Such developmental damage prevents the embryo from growing properly and reaching the blastocyst stage. c‐Abl is also involved in cellular stress responses and interacts with proteins that maintain genetic stability, such as p53, in response to DNA damage. This is essential for the cell's survival and development under conditions of cellular stress (Lamontanara et al. 2014). The loss of c‐Abl can impair cellular responses and DNA repair processes, which may jeopardize genetic stability during embryonic development and cause significant damage to cells at the morula and blastocyst stages. c‐Abl also plays a role in apoptosis and cell survival processes (Zhu and Shore 1996). In the early stages of embryonic development, especially during the morula and blastocyst stages, it is critical for cells to survive properly. The absence of c‐Abl can reduce the cells' ability to survive under stress, leading to increased cell death. This severely impacts the development of the embryo. c‐Abl's genetic regulatory functions play a crucial role during embryonic development (Lamontanara et al. 2014). The absence of c‐Abl prevents the proper regulation of genetic material and can lead to genetic abnormalities in embryonic cells (Lamontanara et al. 2014). This situation disrupts critical processes in the early stages of development, preventing the embryo from reaching the blastocyst stage properly. The observation of prenatal and postnatal mortality in *c‐Abl* homozygous knockout mice indicates that this gene plays critical roles at every stage of development (Li et al. 2000; Qiu et al. 2010; Yildirim and Yaba 2020b). In our study, the damage at the morula and blastocyst stages is also associated with the improper functioning of essential biological processes such as the cell cycle, cellular responses, apoptosis, cell polarization, and genetic stability due to the absence of *c‐Abl*. This can lead to significant disruptions in critical stages of embryonic development and prevent the healthy development of the embryo.

For several fundamental and experimental reasons, studies of the regulation of telomere length in preimplantation development to date are important. With some types of inbred lab rats with incredibly long telomeres, one of them—an embryonic stem (ES) cell derivative—has been successful. It has been demonstrated that telomere length and ES cell pluripotency are correlated (Huang et al. 2011). The mean telomere length in ICM cells in mice is significantly higher than in TE cells (Iqbal et al. 2011; Varela et al. 2011), but in contrast, in TE cells in bovine blastocysts (Iqbal et al. 2011). In the case that mTERT is accepted as an indicator of telomerase activity, the relationship between mTERT and c‐Abl may provide important information for our understanding of the developmental potential of the embryo. Although mTERT has been implicated in oocyte and embryonic development in several species (Martín‐Rivera et al. 1998; Wright 2001), and telomere length may change according to the stage of early embryo development (Turner et al. 2010), there is very little information regarding the interaction of c‐Abl and mTERT during early embryonic development. In the present study, we proposed that c‐Abl may play a role in the regulation of telomerase function in mouse early embryonic development as a result of its interaction with mTERT. Our results suggest that *c‐Abl* may be involved in a regulatory mechanism that controls the transcription of *mTERT*. The relationship between *c‐Abl* and *mTERT* could be part of a mechanism associated with the cell cycle and developmental stages. Moreover, the correlation between these two genes is plausible due to their involvement in the cell cycle and cellular survival. Specifically, *c‐Abl*'s ability to phosphorylate transcription factors and how these factors might affect the genetic expression of *mTERT* could form the basis of this correlation. If *c‐Abl* is involved in a pathway that directly influences the transcription of *mTERT*, the coregulation of these two genes is possible. This correlation can be considered spec. ulative because there is currently no direct evidence of an interaction or mechanism between *c‐Abl* and *mTERT*. Further studies are needed to confirm such a connection, such as testing whether *c‐Abl* binds to the *mTERT* promoter region or investigating in more detail the role of *c‐Abl* in regulating *mTERT* transcription levels

Studies have shown that many molecular mechanisms play a role in the compaction, polarization, and cell fate determination of blastomeres in preimplantation embryo development. Morphological anomalies such as fragmentation, multinucleation, and blastomeres that have problems in the mitotic division are encountered in preimplantation embryos of patients undergoing in vitro fertilization (IVF) treatment (Wong et al. 2010). Although further studies are needed to elucidate the relationship between c‐Abl and mTERT in early embryogenesis, this study suggests that the interaction between c‐Abl and mTERT plays a critical role in compaction and may be essential for maintaining genomic stability up to the point where cell fate determination occurs during preimplantation embryo development. The involvement of c‐Abl and mTERT in earlier stages, before the determination of cell fate, could ensure the preservation of genomic stability required for successful developmental progression. The present study highlights the necessity of enhancing comprehension of the role of the c‐Abl protein and its association with transcriptional and posttranslational controls throughout the initial stages of embryonic development. In conclusion, c‐Abl and mTERT have an essential regulative function during preimplantation embryo development for addressing underlying molecular mechanisms of early embryogenesis. The primary aim of this study is to highlight the critical roles of mTERT and c‐Abl tyrosine kinase genes in maintaining genomic stability during the early stages of embryonic development, and to demonstrate how abnormalities in these genes can disrupt the healthy development of embryonic cells, particularly during vital developmental stages such as compaction. Additionally, this study seeks to gain a deeper understanding of the fundamental mechanisms of embryonic development and contribute to the identification of new biological targets for potential therapeutic strategies by focusing on these mechanisms. In this way, a more comprehensive perspective on how to ensure the proper functioning of genetic and cellular processes for successful embryonic development is presented.

## Author Contributions

**Ecem Yildirim:** conceptualization, methodology, investigation, formal analysis, visualization, writing – original draft. **Tugce Onel:** investigation, methodology, visualization, formal analysis. **Aylin Yaba:** conceptualization, methodology, supervision, validation, investigation, funding acquisition, writing – original draft, writing – review and editing.

## Disclosures

The authors have nothing to report.

## Ethics Statement

All animals used in this study were obtained from the Yeditepe University Faculty of Medicine Experimental Research Center (YUDETAM) and all the experimental procedures have been approved by the Yeditepe University Ethical Committee. This manuscript complies with the ARRIVE guidelines.

## Consent

The authors have nothing to report.

## Conflicts of Interest

The authors declare no conflicts of interest.

## Data Availability

Data sharing is not applicable to this article as no new data were created or analyzed in this study.
